# Short-Wavelength Infrared Hyperspectral Imaging and Spectral Unmixing Techniques for Detection and Distribution of Pesticide Residues on Edible Perilla Leaves

**DOI:** 10.3390/foods14162864

**Published:** 2025-08-18

**Authors:** Dennis Semyalo, Rahul Joshi, Yena Kim, Emmanuel Omia, Lorna Bridget Alal, Moon S. Kim, Insuck Baek, Byoung-Kwan Cho

**Affiliations:** 1 Department of Smart Agricultural Systems, College of Agricultural and Life Science, Chungnam National University, Yuseong-gu, Daejeon 34134, Republic of Korea; semyalod@gmail.com (D.S.); lorna.b.alal12@gmail.com (L.B.A.); 2 Department of Agricultural Machinery Engineering, College of Agricultural and Life Science, Chungnam National University, Yuseong-gu, Daejeon 34134, Republic of Korea; rahul.joshi98@yahoo.com (R.J.); kyn6874@naver.com (Y.K.); deusviam@gmail.com (E.O.); 3 Environmental Microbial and Food Safety Laboratory, Agricultural Research Service, United States Department of Agriculture, Powder Mill Road, BARC-East, Bldg 303, Beltsville, MD 20705, USA

**Keywords:** pesticide residue analysis, hyperspectral imaging, spectral unmixing, food safety, chemometrics, multicurve resolution-alternating least squares

## Abstract

Pesticide residue analysis of agricultural produce is vital because of associated health concerns, highlighting the need for effective non-destructive techniques. This study introduces a method that combines short-wavelength infrared hyperspectral imaging with spectral unmixing to detect chlorfenapyr and azoxystrobin residues on perilla leaves. Sixty-six leaves were treated with pesticides at concentrations between 0 and 0.06%. The study utilized multicurve resolution-alternating least squares (MCR-ALS), a spectral unmixing method, to identify and visualize the distribution of pesticide residues. This technique achieved lack-of-fit values of 1.03% and 1.78%, with an explained variance of 99% for both pesticides. Furthermore, a quantitative model was developed that integrates insights from MCR-ALS with Gaussian process regression to estimate chlorfenapyr residue concentrations, resulting in a root mean square error of double cross-validation (RMSEV) of 0.0012% and a double cross-validation coefficient of determination (R^2^v) of 0.99. Compared to other chemometric approaches, such as partial least squares regression and support vector regression, the proposed integrated method decreased RMSEV by 67.57% and improved R^2^v by 2.06%. The combination of hyperspectral imaging with spectral unmixing offers advancements in the real-time monitoring of agricultural product safety, supporting the delivery of high-quality fresh vegetables to consumers.

## 1. Introduction

Perilla leaves (*Perilla frutescens*) hold significant cultural, medicinal, and culinary importance in South Korea and other Asian communities such as Japan, China, India, Thailand, and Vietnam [1]. In recent years, the popularity of perilla leaves has extended to European and North American markets, driven by an increasing awareness of their economic potential and health benefits [2]. As one of the most widely consumed salad greens in Korea, they serve as an ingredient in traditional dishes such as *ssam* (wrap), *kimchi*, soups, and sushi [3]. Perilla has also been important to traditional medicine due to its rich phytochemical profile with anti-inflammatory, antioxidant, and antimicrobial properties [1,4]. The use of pesticides, including fungicides, insecticides, and herbicides, remains a common agricultural practice to mitigate crop losses from pests and diseases, thereby ensuring higher yields [5]. However, excessive or improper application often leads to persistent pesticide residues on agricultural produce, posing significant risks to human health [2]. An estimated three million people globally suffered from pesticide poisoning in 2022, with mortality rates reaching approximately 20% [6]. Prolonged exposure to pesticide residues causes severe health conditions, including cardiovascular diseases and cancer [7,8]. In recent years, growing consumer awareness of these risks has led to stricter regulations on permissible pesticide residue levels in agricultural products [7]. Despite these measures, the misuse of pesticides during the cultivation of crops, including perilla leaves, remains a significant concern [3]. This underscores the need for advanced detection methods to ensure food safety and mitigate health hazards [2,6].

Conventional methods for detecting pesticide residues in fruits and vegetables include high-performance liquid chromatography (HPLC) [9], gas chromatography (GC) [10], liquid chromatography-mass spectrometry (LC–MS) [11], gas chromatography-mass spectrometry (GC–MS) [12], supercritical fluid chromatography (SFC) [13], and capillary electrophoresis (CE) [14]. While these techniques offer high accuracy, they are often costly, require complex sample preparation procedures, demand specialized personnel for operation, and cause irreversible damage to the samples [5]. There is a need to develop advanced, non-destructive methods for identifying pesticide residues on agricultural produce.

Hyperspectral imaging (HSI) has gained prominence in various agricultural applications, especially in evaluating the safety and quality of fruits and vegetables [15,16]. HSI provides the ability to capture detailed spatial and spectral information simultaneously, making it highly suitable for nondestructively assessing pesticide residues on agricultural produce [17,18]. Recent studies have demonstrated the effectiveness of HSI in detecting pesticide residues on agricultural produce, including Hami melons, achieving a classification accuracy exceeding 90% using t-distribution honey badger algorithm-extreme learning machine (tHBA-ELM) [19]. However, to our knowledge, this is the first study to combine HSI with advanced spectral unmixing methodologies for pesticide residue detection, specifically targeting edible perilla leaves. Spectral unmixing enhances HSI’s capabilities by decomposing mixed spectral signals into pure component spectral signatures and their corresponding concentration profiles, even without requiring prior information [20,21,22]. Multivariate curve resolution-alternating least squares (MCR-ALS), a chemometric technique commonly used in spectral unmixing, enables the separation of overlapping spectral data, facilitating accurate identification of pesticide residues in agricultural produce [20,23,24]. This study proposes a rapid and nondestructive approach that leverages hyperspectral imaging technology combined with spectral unmixing. Therefore, the study aimed to investigate the nondestructive detection and distribution of various pesticide residues on the surface of perilla leaves using spectral unmixing and hyperspectral imaging. The integration of HSI with spectral unmixing is a powerful approach for detecting pesticide residues, offering a more efficient and comprehensive solution for ensuring food safety and quality control in agriculture.

## 2. Materials and Methods

### 2.1. Sample Preparation

This feasibility study was conducted in a laboratory setting to simulate the real-world pesticide application process on Perilla leaves. Perilla leaves were procured from a local market in Daejeon, South Korea, and a total of 66 fresh, undamaged leaves were selected for the experiment. Two commonly applied pesticides in South Korea, chlorfenapyr and azoxystrobin, were selected for this research based on their widespread use in fruit and vegetable farming [25]. Chlorfenapyr (5% active ingredient, Taejun aggrotech, Seongnam, South Korea) and azoxystrobin (21.7% active ingredient, Syngenta, Basel, Switzerland) pesticides were acquired in liquid form from a licensed local agrochemical supplier. Chlorfenapyr and azoxystrobin pesticides were characterized under emulsifiable concentrate (EC) and suspension concentrate (SC) commercial formulations, respectively. The pure reference standards (95–100% purity, Sigma-Aldrich, St. Louis, Missouri, United States) were obtained for spectral validation. Safety protocols, including the use of gloves and masks, were strictly followed during pesticide handling. In addition, the entire experiment was conducted inside a chemical fume hood to prevent any harm that could occur during the preparation of different pesticide concentrations.

The pesticides were diluted with distilled water to a concentration of 0.05% (original active ingredient), according to the recommended dosage for farmers [26]. The farmers can apply this dosage 2 times, 3 days before harvest. This dilution is based on the efficacy of these pesticides in protecting perilla leaves against pests and diseases. Additionally, solutions with concentrations of 0.06%, 0.04%, 0.02%, and 0% (control) were prepared for the quantitative estimation of chlorfenapyr residues across a realistic range, especially at lower levels. Perilla leaf samples were placed on a tray before pesticide application, as illustrated in Figure S1. The pesticide solutions were applied onto the Perilla leaf surfaces using spray cans [27]. The sprayed leaf samples were placed in a drying chamber maintained at an optimal storage temperature of 6.5 °C and 50% relative humidity for approximately 24 h. These conditions were selected based on recommendations for commercial postharvest handling of perilla leaves, which minimize physiological disorders and deterioration [28,29]. The tray with the sprayed samples was covered with a perforated polythene bag to allow air circulation while preventing leaf deformation. Once the perilla leaf surfaces were free of residual liquid, short-wavelength infrared (SWIR) hyperspectral imaging data acquisition was conducted.

### 2.2. Hyperspectral Imaging System

**Figure 1:**
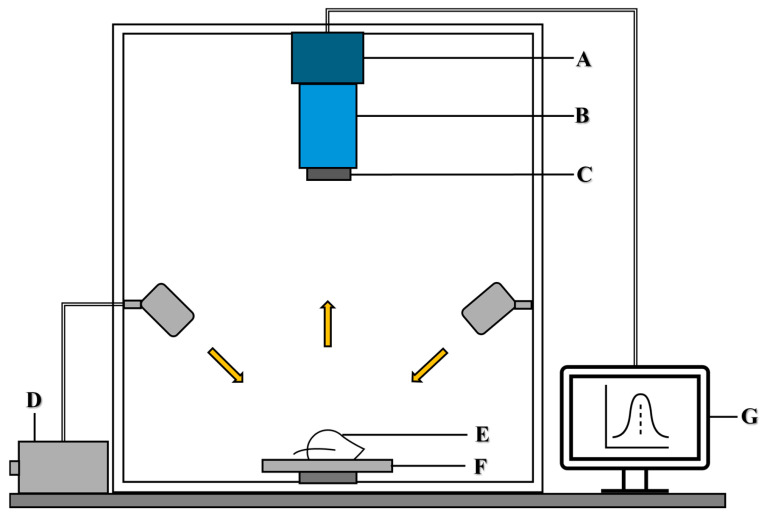
Short-wave infrared hyperspectral imaging system for spectral data collection. A: camera, B: spectrograph, C: lens, D: light source, E: leaf sample, F: translation stage, G: computer.

This study utilized a short-wave infrared (SWIR) hyperspectral imaging (HSI) system to identify, detect, and quantify the distribution of pesticide residues on perilla leaves. The SWIR HSI system comprised core components including a spectrograph (Headwall Photonics, Fitchburg, MA, USA) with a spectral resolution of 5.876 nm and covering 894–2504 nm wavelengths, and a mercury cadmium telluride detector array (Xeva-2.5-320, Xenics, Belgium) with a camera resolution of 320 × 256 pixels, a 25 mm f/1.4 objective lens (OB-SWIR25/1.4, P/N C0808.010). Sample illumination was achieved using six fiber-optic-coupled 100 W tungsten-halogen lamps (Light Bank, Ushio Inc., Tokyo, Japan) arranged to ensure uniform spectral acquisition. A DC motor-controlled translation stage precisely advanced mixed samples through the field of view of the camera during HSI data acquisition. The detailed specifications of the SWIR HSI system are presented in Table S1. A schematic representation of the main components of the SWIR HSI system is provided in Figure 1.

The perilla leaves with pesticide residues were collected from the drying chamber and placed on a black background plate above the translation stage of the SWIR HSI system. During data acquisition, the mixed samples were scanned at an optimal speed of 5.496 mm/s with an exposure time of 0.043 s. The motor-driven translation stage advanced the conveyor unit through the camera’s field of view (FOV), enabling line-by-line spectral acquisition. The data were recorded as a three-dimensional hypercube, capturing spectral and spatial information. To ensure accurate calibration, a white reference image was captured using a standard white Teflon, while a dark reference image was obtained by turning off the light source and ensuring no light reached the camera detector. The captured hyperspectral image of the mixed samples was corrected for any distortions or noise introduced by the imaging system and environmental conditions. This included the dark current correction and white reference correction as shown in Equation (1) [30,31].

(1) $$I_{cor}=\frac{I_{raw}-I_{dark}}{I_{white}-I_{dark}}$$

where $I_{cor}$ is the corrected intensity, $I_{raw}$ is the raw captured intensity measured from the mixed sample, $I_{dark}$ is the intensity measured with no light, and $I_{white}$ is the intensity measured from a white reference standard [32]. After calibration, regions of interest (ROIs) within the corrected hypercube were selected. The ROI of the calibrated hyperspectral image was unfolded to create a data matrix, D, which was utilized during spectral unmixing analysis of pesticide residues on perilla leaves.

### 2.3. Multivariate Curve Resolution-Alternating Least Squares for Pesticide Residue Analysis

Multivariate curve resolution-alternating least squares (MCR-ALS) is a spectral unmixing approach that extracts the pure spectral signatures of constituents and determines their spatial distribution within complex mixture samples or measurements. It is fundamentally based on a bilinear model, as represented in Equation (2)

(2) $$D=CS^{T}+E$$

where D is a 2D matrix built by unfolding the original hypercube, C is a distribution matrix, $S^{T}$ is the matrix containing pure spectra information, and E is the matrix expressing error or variance unexplained by the bilinear model [33]. This method decomposes hyperspectral images into distinct spectral profiles and their corresponding distributions for each compound present in the sample [34]. As an iterative optimization algorithm, MCR-ALS refines initial estimates of either spectral or concentration profiles by alternatingly minimizing residuals under user-defined constraints until the solution satisfies predefined convergence criteria [23].

The MCR-ALS technique was utilized to identify, detect, and visualize the distribution of chlorfenapyr and azoxystrobin pesticide residues on perilla leaves, using a sample concentration of 0.05%. For spectral unmixing of chlorfenapyr residues, the initial estimates of the spectral matrix (S^T^) were derived from pure reference spectra. In contrast, the initial spectral estimates for azoxystrobin residues were determined using the simple-to-use self-modeling mixture analysis (SIMPLISMA) algorithm, a pure variable selection method. During optimization, non-negativity constraints were applied to both the spectral and distribution matrices to ensure realistic results. MCR-ALS models with 2, 3, 4, and 5 components were also tested during the optimization process. The two-component model was selected, as additional components beyond two showed no meaningful distribution in the resolved maps. This aligns with the expected pure components of mixed samples containing pesticide residue and perilla leaf. The optimization was performed using a convergence criterion of 0.1% [23] change between iterations and a maximum of 10,000 iterations. MCR-ALS analysis was performed using MCR-ALS GUI 2.0 [21], which can be downloaded at mcrals.info. The performance of MCR-ALS for pesticide residue analysis was assessed using the percent lack of fit (LOF) defined in Equation (3), the percentage of variance explained ($R^{2}$) in Equation (4), and the visualization of pesticide residue distributions on perilla leaves [33].

(3) $$\%LOF=100\sqrt{\frac{\sum_{i,j}{e_{i,j}}^{2}}{\sum_{i,j}{d_{i,j}}^{2}}}$$

(4) $$R^{2}\left(\%\right)=100\times \frac{\sum_{i,j}{d_{i,j}}^{2}-\sum_{i,j}{e_{i,j}}^{2}}{\sum_{i,j}{d_{i,j}}^{2}}$$

where LOF is the lack of fit, $R^{2}$ is the percentage of variance explained, $d_{i,j}$ is an element of the data matrix D, and $e_{i,j}$ is the related residual.

### 2.4. Quantitative Model Development for Pesticide Residue Estimation

The quantitative model development for estimating the concentrations of chlorfenapyr residue on perilla leaves was based on the prepared solutions of 0.06, 0.04, 0.02, and 0%. For each leaf, hyperspectral images were acquired, and two regions of interest (ROIs) were extracted, representing half of the leaf each. Firstly, spectral unmixing with MCR-ALS was performed. The output from MCR-ALS provides spectral and distribution profiles at each concentration for both the chlorfenapyr residue and perilla leaf. Based on the distribution profile of the chlorfenapyr residue, the spatial areas and positions with the pesticide residue were mapped and were visible. These maps highlighted areas where the pesticide was present, allowing extraction and averaging of spectral data corresponding to residue-containing regions. The regions with the highest amount of chlorfenapyr residue in the reconstructed distribution profile were used to extract the spectral data for the development of the quantitative model. Each leaf thus contributed two average spectra, forming the dataset for regression modeling. This resulted in 120 spectral observations for training the Gaussian process regression (GPR) model. Therefore, the insights from the distribution profiles obtained by MCR-ALS were combined with Gaussian process regression (GPR) to predict the chlorfenapyr pesticide residue concentrations on perilla leaves. The extracted spectral data were randomly split into two groups: one for calibration, comprising 70% of the data, and the other for the test set to be used during double cross-validation, consisting of 30% of the data. This was done while maintaining the balance between the mean and standard deviation of the reference data [30,35]. The test set for double cross-validation was not used during the model development. The statistics for the extracted spectra data, calibration, and test sets are illustrated in Table 1. A wavelength range of 918–2058 nm was utilized during analysis. The analysis in this study was performed in the MATLAB environment (2021a, The MathWorks, Natick, MA, USA).

GPR is a non-parametric machine learning approach that defines a distribution over functions and uses this distribution to make predictions [36]. Consider a dataset $D=\left\{\left(x_{i},y_{i}\right)|i=1,2,\ldots ,m\right\}=\left(X,y\right)$, where each input $x_{i}\in R^{d}$ represents an observation (for example, a spectral measurement) comprising d variables (such as spectral wavelengths), and the scalar $y_{i}$ corresponds to the measured response (e.g., pesticide residue concentration) associated with $x_{i}$. The dataset, D is represented by the matrix, $X=\left[x_{1},x_{2},\ldots ,x_{m}\right]$ and the vector, $y={\left[y_{1},y_{2},\ldots ,y_{m}\right]}^{T}$. Within the Gaussian process framework, each response y is modeled as in Equation (5) [37]

(5) $$y=g\left(x\right)+\eta$$

where g (∙) denotes a latent function following a Gaussian process, illustrated in Equation (6)

(6) $$g\left(x\right)\sim GP\left(0,k\left(x,x^{’}\right)\right)$$

and η represents an additive Gaussian noise shown in Equation (7)

(7) $$\eta \sim N\left({\sigma^{2}}_{m}\right)$$

where x and y denote any single observation and its corresponding response, and $k\left(x,x^{’}\right)$ represents the kernel function that defines the covariance between any two points x and x’. The assumption in Equation (6) indicates that the latent function g (∙) is controlled by a Gaussian process (GP). This process is characterized by a zero mean function and a covariance function $k\left(x,x^{’}\right)$, while the noise term η is assumed to be normally distributed with zero mean and variance ${\sigma^{2}}_{m}$ [36]. The choice of kernel function is crucial in GPR as it defines the type of functions that can be learned [38]. In this study, several kernel functions were evaluated, including squared exponential (SE), Matérn kernel with different smoothness parameters (ν = 3/2, 5/2), and rational quadratic (RQ). Hyperparameter optimization was performed using maximum likelihood estimation (MLE), with a 5-fold cross-validation approach to prevent overfitting. The optimal kernel function was selected based on double cross-validation performance and model complexity. The assessment metrics for the developed quantitative model included the coefficient of determination (R^2^v) and the root mean square error (RMSEV) of double cross-validation for the pesticide residue concentration. The estimation of chlorfenapyr residue concentration on perilla leaves was also performed with other quantitative techniques such as partial least squares regression (PLSR) and support vector regression (SVR).

The limit of detection (LOD) is a critical parameter in the validation of analytical methods and is highlighted in various regulatory guidelines. LOD refers to the lowest concentration of an analyte that can be reliably detected by the method, with a certain degree of confidence [39]. In this study, the LOD for chlorfenapyr residues was established using the standard deviation and slope method, in accordance with recommendations from the International Council for Harmonisation (ICH) [40,41,42]. This method determines LOD using Equation (8)

(8) $$LOD=\frac{3.3\rho }{S}$$

where ρ represents the residual standard deviation, and S is the slope of the regression line. The residual standard deviation is obtained from regression analysis. Recent research has shown that this approach yields a reliable estimate of LOD when applied to regression-based methods [39]. This ensures that the analytical technique is dependable for detecting low levels of chlorfenapyr residue in agricultural produce, aligning with established international guidelines.

## 3. Results and Discussion

### 3.1. Spectra of Perilla Leaf and Mixed Samples with Pesticide Residues

**Figure 2:**
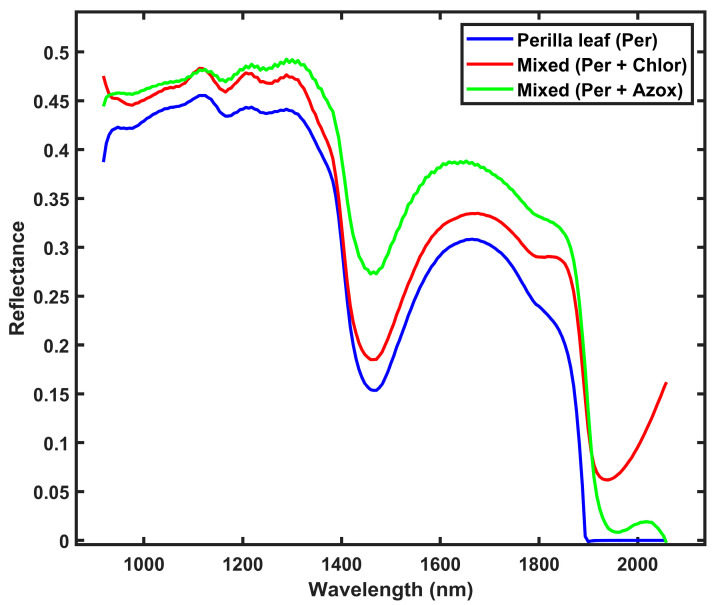
Mean spectra of perilla leaf and mixed samples with pesticide residues. Per: Perilla, Chlor: chlorfenapyr residue, and Azox: azoxystrobin residue.

The spectral characteristics of perilla leaf samples with and without pesticide residues reveal patterns in the SWIR region, as illustrated in Figure 2. The untreated perilla leaf samples exhibit lower reflectance values compared to samples treated with either chlorfenapyr or azoxystrobin throughout the SWIR spectrum. The enhanced reflectance observed in pesticide-treated leaves can be attributed to the chemical composition of the pesticides and their interaction with the leaf surface structure. The untreated perilla leaf samples exhibit characteristic absorption and reflectance peaks at 1123, 1170, 1300, 1450, and 1652 nm. Perilla leaves treated with chlorfenapyr pesticide show spectral characteristics with absorption and reflectance peaks at 1112, 1206, 1450, 1664, 1852, and 1940 nm. Samples treated with azoxystrobin exhibit the highest overall reflectance, with wavelength peaks at 1217, 1450, 1630, and 2030 nm.

The SWIR region is sensitive to vibrational modes of molecular bonds, especially those involving O-H, C-H, and N-H groups [27,43]. The absorption peak at 1450 nm present in all samples is associated with the first overtone of O-H stretching vibrations in water molecules [38,44,45]. However, the intensity of this peak varies between samples due to the interaction of pesticide molecules with leaf surface water and distilled water used to make the pesticide residue solution. The spectral differences between untreated and pesticide-treated perilla leaves occur in several regions, especially beyond 1900 nm. After 1900 nm, the spectral patterns diverge, with untreated samples showing near-zero reflectance, while both pesticide-treated samples maintain some reflectance. The observed spectral differences between untreated and pesticide-treated perilla leaves provide a basis for developing non-destructive methods for the detection of pesticide residues using spectral unmixing and SWIR hyperspectral imaging.

### 3.2. Detection and Distribution of Chlorfenapyr Residues on Perilla Leaves

**Figure 3:**
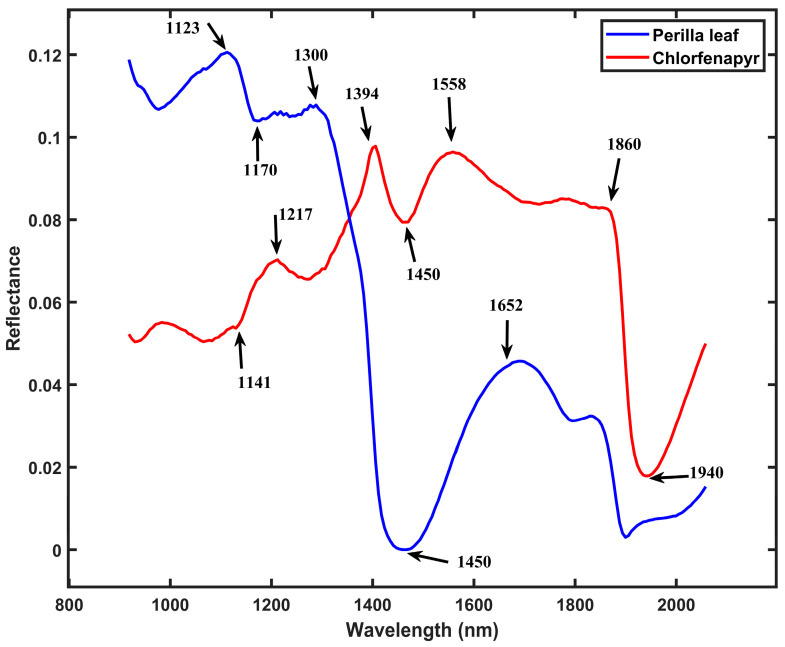
The recovered pure spectral profiles of chlorfenapyr residue and perilla leaf using the multivariate curve resolution-alternating least squares (MCR-ALS).

Multivariate curve resolution-alternating least squares (MCR-ALS) was utilized as a chemometric technique to resolve the spectral data of mixed perilla leaf and pesticide samples into their pure components. The MCR-ALS algorithm identified and extracted the spectral profiles of both pure perilla leaf and chlorfenapyr pesticide residue from the hyperspectral data matrix, as shown in Figure 3. This spectral unmixing technique achieved a lack of fit (LOF) of 1.03% and an explained variance of 99%, as illustrated in Table 2. The unmixing performance indicates a good representation of the original data matrix of mixed leaf samples using the bilinear decomposition model. The alternating least squares process converged after an average of 1411 iterations in separating the overlapping spectral features of the mixed leaf samples in the SWIR region. The recovered pure perilla leaf spectrum exhibited characteristic reflectance and absorption peaks at 1450, 1123, 1170, 1300, and 1652 nm. The resolved pure chlorfenapyr spectrum showed reflectance and absorption peaks in 1940, 1394, 1217, 1558, 1860, and 1141 nm. The resolution of the pure chlorfenapyr spectrum could have been affected by interfering signals from secondary ingredients present in the purchased liquid pesticide formulation, including emulsifiers and solvents.

The differentiation between pure perilla leaf and pure chlorfenapyr pesticide residue through MCR-ALS is based on their molecular structures and chemical compositions. The specific wavelength peaks identified in each pure component correspond to molecular bonds and functional groups that absorb or reflect infrared radiation at characteristic frequencies. The spectral features of pure perilla leaf are characteristic of plant tissue and are primarily attributed to the vibrational modes of key biochemical constituents, such as water, flavonoids, phenolic acids, and carbohydrates [1,46,47]. The absorption peak at 1450 nm is associated with O-H stretching vibrations in water molecules, such as plant tissue water content [38,44,45]. The wavelengths at 1170 nm are likely associated with the second overtone of C-H vibrations from carbohydrates [44,48,49]. The SWIR pure spectral profile of chlorfenapyr pesticide [4-bromo-2-(4-chlorophenyl)-1-(ethoxymethyl)-5(trifluoromethyl)-1H-pyrrole-3-carbonitrile] exhibits absorption and reflectance features that can be attributed to its molecular structure (C15H11BrClF3N2O) [50,51]. The absorption features at 1394 nm likely correlate with C−H combination vibrations, while the absorption at 1141 can be attributed to C-H stretching overtones in the aromatic ring [44]. The wavelength feature at 1860 nm represents overtones of vibrations from the molecule’s halogenated components that influence the pesticide’s spectral profile. Specifically, it corresponds to the sixth overtone of C−C1 stretching [44,50]. The separation of these spectral components demonstrates that MCR-ALS can effectively manage spectral overlap in hyperspectral imaging data, providing a non-destructive method for pesticide residue detection on agricultural products. The application of this chemometric approach to hyperspectral imaging represents an advancement over traditional methods that require time-consuming sample preparation and destructive analytical procedures.

**Figure 4:**
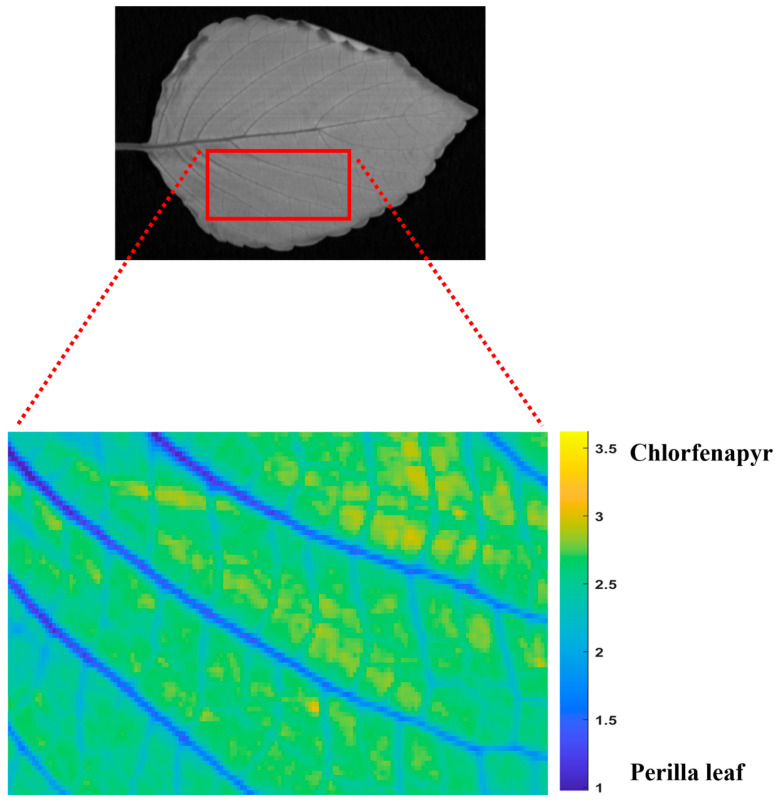
The distribution of chlorfenapyr residues using MCR-ALS.

The spatial distribution of chlorfenapyr residues on perilla leaves was visualized using MCR-ALS analysis of hyperspectral imaging data, as illustrated in Figure 4. The distribution mapping reveals a pattern where chlorfenapyr residues and perilla leaf components are distributed across the leaf surface with an average proportion of 48.63% and 51.37%, respectively. The closely equal distribution demonstrates the retention of the pesticide on the leaf surface. The MCR-ALS analysis demonstrates that the pesticide residues accumulated in the lamina of the perilla leaf rather than along the veins or margins due to the morphological characteristics of perilla leaves. The spatial heterogeneity in the distribution maps reveals that pesticide deposition is not uniform across the leaf surface, with some regions showing higher concentrations than others. The chlorfenapyr distribution patterns determined by using MCR-ALS could inform more targeted analysis strategies for quantification of pesticide residue concentration using machine learning techniques. The MCR-ALS chemometric approach used in this study offers advantages over traditional pesticide analysis methods, as it enables non-destructive, real-time visualization of pesticide residues while simultaneously separating the spectral profiles of the pesticide from the plant matrix.

### 3.3. Detection and Distribution of Azoxystrobin Residues on Perilla Leaves

**Figure 5:**
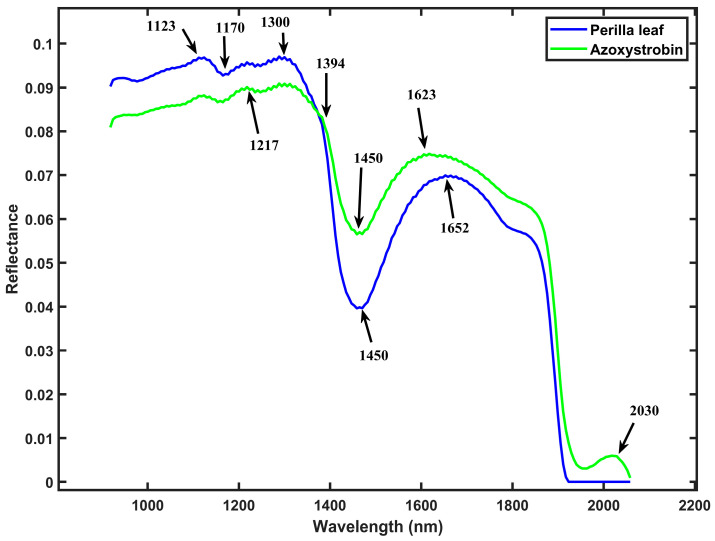
The resolved pure spectral profile of azoxystrobin residue and perilla leaf using MCR-ALS.

The pure spectral profiles of azoxystrobin residue and perilla leaf were resolved using the MCR-ALS method, as illustrated in Figure 5. This chemometric approach enabled the decomposition of the mixed leaf spectra into their pure components, achieving a lack of fit (LOF) of 1.78% and an explained variance of 99%, as presented in Table 2. The MCR-ALS resolution of azoxystrobin and perilla leaf achieved convergence within an average of 12 iterations, which is faster compared to the chlorfenapyr pesticide residue resolution. The recovered pure perilla leaf spectrum exhibited reflectance and absorption peaks at 1450, 1123, 1170, 1300, and 1652 nm. These wavelengths are similar to the pure perilla leaf peaks recovered in the previous section during the resolution of mixed leaf samples with chlorfenapyr residue. The resolved pure azoxystrobin spectrum showed characteristic reflectance and absorption peaks in 2030, 1623, 1394, and 1217 nm. The resolution of the pure azoxystrobin spectrum may have been affected by interference from other ingredients present in the purchased liquid pesticide, including wetting agents, antifreeze, thickeners, and bulking agents. Some resolved reflectance and absorption peaks for azoxystrobin showed similarities with those identified for chlorfenapyr in the previous analysis, due to common chemical parts and molecular structures shared between these agrochemicals.

The resolution of pure spectral components through MCR-ALS provides valuable insights into the specific wavelength regions that identify each material. For the azoxystrobin spectrum, the identified reflectance and absorption peaks correlate with the chemical structure of this fungicide. The resolved peak at 1217 nm likely corresponds to the second overtones of C-H stretching vibrations in the azoxystrobin molecule [44]. The 1394 nm feature can be attributed to the C-H combination bands, while the 1623 nm peak likely represents the first overtones of C-H stretching vibrations present in their chemical structure [44]. The unique 2030 nm peak corresponds to the second overtones involving C=O stretching vibrations, a chemical structural feature present in azoxystrobin [44,52,53]. The spectral resolution achieved with the performance metrics demonstrates the effectiveness of MCR-ALS in extracting meaningful chemical information from hyperspectral data.

**Figure 6:**
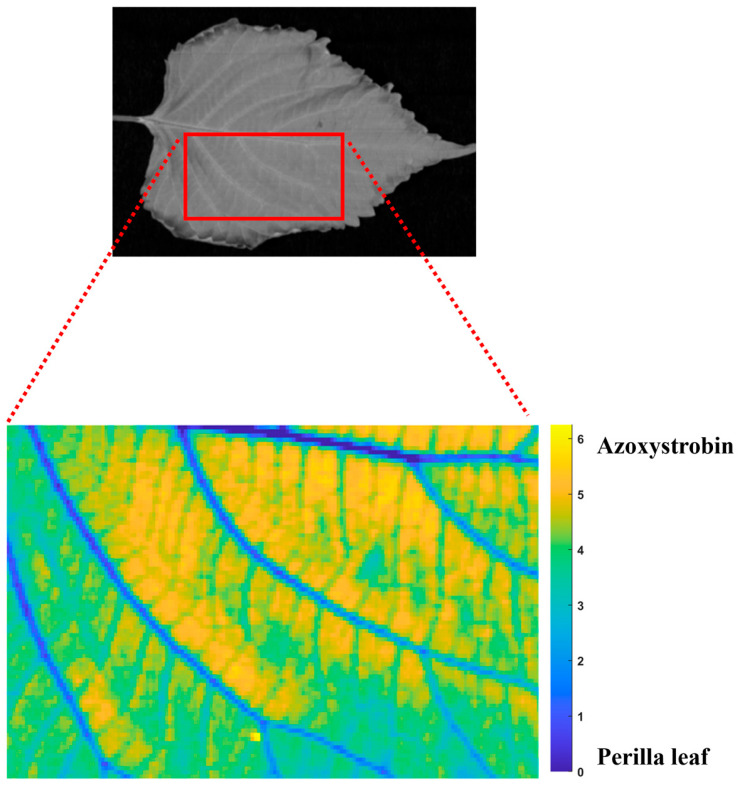
The distribution of azoxystrobin residues using MCR-ALS.

The spatial distribution of azoxystrobin residues on perilla leaves was visualized using MCR-ALS analysis, as illustrated in Figure 6. The distribution mapping revealed a pattern where azoxystrobin residues accounted for an average proportion of 70.51% of the analyzed area, while perilla leaf components comprised only 29.49%. The proportion of azoxystrobin compared to plant material indicates retention and surface coverage of fungicide. This enhances its efficacy against target pathogens but also raises considerations about residue management and food safety. The MCR-ALS visualization reveals that azoxystrobin distributes with heterogeneity across the leaf surface. Previous studies, such as that by [54] found that azoxystrobin concentrates mostly in the outer bracts when applied to artichokes. The advanced visualization technique utilized in this study represents an improvement over traditional pesticide residue analysis methods, as it provides spatial distribution information that cannot be obtained through conventional extraction-based approaches.

### 3.4. Estimation Model for Chlorfenapyr Residue Concentration on Perilla Leaves

**Figure 7:**
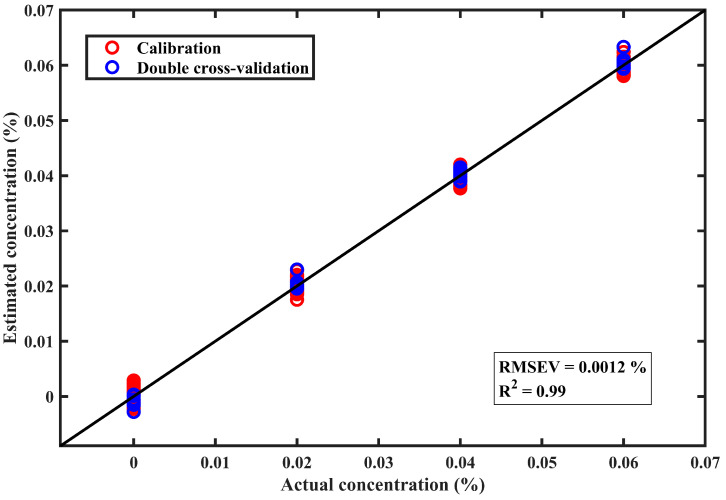
The calibration and double cross-validation plot for chlorfenapyr residue concentration on perilla leaves using the multivariate curve resolution-alternating least squares combined with Gaussian process regression. R^2^ is the coefficient of determination, and RMSEV is the root mean square error of double cross-validation. The number of observation spectra was 120.

A quantitative model combining MCR-ALS with Gaussian Process Regression (GPR) was developed to estimate chlorfenapyr residue concentrations on perilla leaves. This integrated approach leverages the strengths of both techniques, with MCR-ALS providing spectral unmixing capabilities and GPR offering robust predictive modeling. The model was developed using regions with the highest amount of chlorfenapyr residue in the reconstructed distribution profile. As illustrated in Table 3 and Figure 7, the calibration performance of the model achieved a coefficient of determination (R^2^c) of 0.99 and a root mean square error of calibration (RMSEC) of 0.0013%. The validation performance, shown in Table 3 and Figure 7, achieved a double cross-validation coefficient of determination (R^2^v) of 0.99 and a root mean square error of double cross-validation (RMSEV) of 0.0012%. The limit of detection (LOD) for chlorfenapyr residue was computed, yielding a concentration of 0.0042% (approximately 2.1 ppm). Therefore, the proposed approach detects lower concentrations below the maximum residue limit for chlorfenapyr residue (approximately 7 ppm) [55]. This indicates that the developed technique can detect chlorfenapyr at low concentrations and is suitable for quality and safety inspection of pesticide residues on agricultural produce.

The study also revealed insights into the relationship between pesticide residue concentration and MCR-ALS algorithm performance. The mixed leaf samples with high chlorfenapyr residue concentrations were found to be easily detected, requiring fewer iterations for the MCR-ALS algorithm to converge on a solution. In contrast, the mixed leaf samples with lower chlorfenapyr residue concentrations presented detection challenges, necessitating more iterations for the MCR-ALS algorithm to achieve convergence. Therefore, the lower the pesticide residue concentration, the more iterations were required during spectral unmixing with MCR-ALS. The integrated MCR-ALS and GPR modeling approach represents an advancement in the non-destructive quantification of pesticide residues on agricultural products.

**Figure 8:**
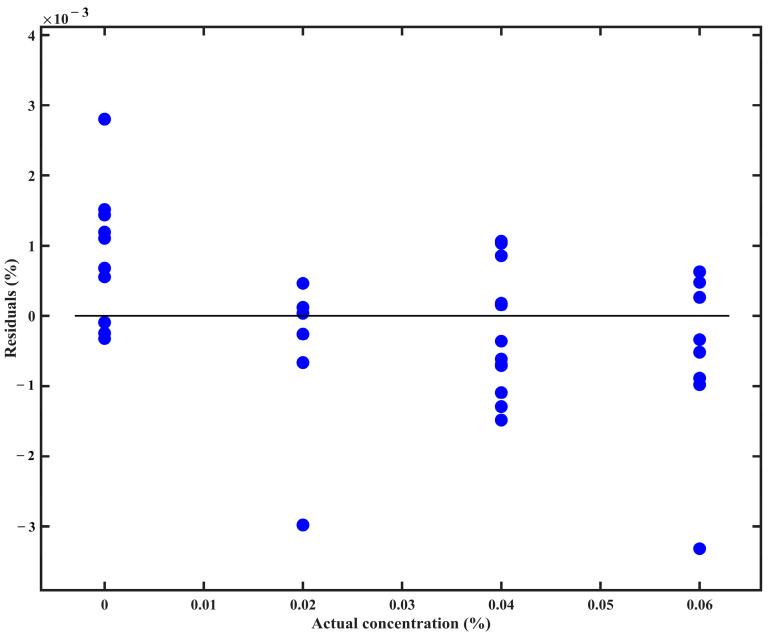
Double cross-validation residual plot for chlorfenapyr residue concentration on perilla leaves using the multivariate curve resolution-alternating least squares combined with Gaussian process regression.

A residual plot for the GPR model predictions, evaluated using the double cross-validation set, is shown in Figure 8. Most residuals are distributed around zero with no trend across the concentration range, supporting the assumption of homoscedasticity for the model. The residuals are generally small in magnitude and are tightly clustered near zero, with only a few mild outliers. These results demonstrate that the model provides robust and reliable predictions for chlorfenapyr residue concentration on perilla leaves.

### 3.5. Comparison with Other Chemometric Techniques for Chlorfenapyr Residue Concentration on Perilla Leaves

The integration of spectral unmixing techniques with regression methods represents an advancement in pesticide residue detection and quantification in agricultural produce. Spectral unmixing enhances hyperspectral imaging (HSI) capabilities by decomposing mixed spectral signals into pure component spectral signatures and their corresponding distribution profiles [20,21,22]. MCR-ALS provided useful distribution maps for visualizing residues, but it was not effective as a standalone quantitative technique for pesticide residue concentration estimation. Therefore, the insights derived from MCR-ALS were integrated with Gaussian process regression (GPR) to enhance the regression analysis capabilities for chlorfenapyr residue concentration estimation on perilla leaves. As demonstrated in Table 3, the proposed MCR-ALS–GPR approach outperformed conventional chemometric techniques, including partial least squares regression (PLSR) and support vector regression (SVR) across all evaluated performance metrics. The integrated approach achieved higher coefficient of determination values and lower root mean squared errors. The approach improved the R^2^v by 2.06% and led to a 67.57% reduction in the RMSEV when compared with all the other alternative techniques. The performance of the MCR-ALS–GPR integrated approach can be attributed to MCR-ALS’s ability to effectively separate overlapping spectral signatures, combined with GPR’s nonlinear modeling capabilities that capture relationships between spectral features and pesticide concentrations. MCR-ALS enables the effective separation of overlapping spectral data by iteratively resolving distribution and spectral profiles from multivariate datasets [20,24], which conventional chemometric techniques struggle with. This capability is valuable in agricultural and environmental analytical chemistry, where complex sample matrices often exhibit spectral interference.

### 3.6. Visualization of Chlorfenapyr Residue Concentrations on Perilla Leaves

**Figure 9:**
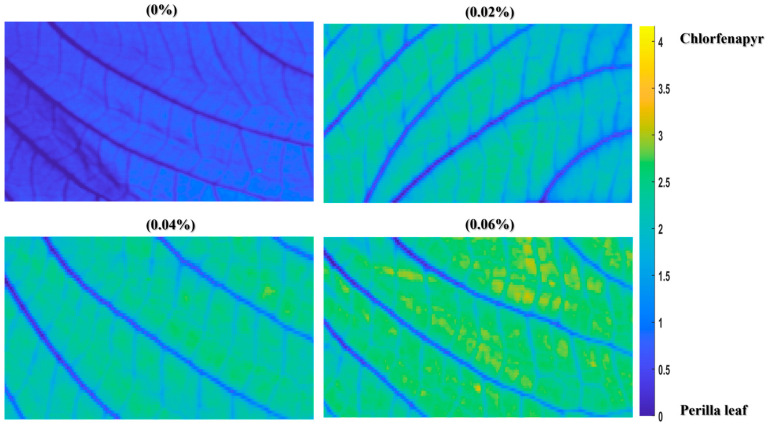
The distribution of chlorfenapyr residue concentrations using MCR-ALS.

The visualization of chlorfenapyr residue concentrations on perilla leaf through MCR-ALS analysis provides a spatial representation of pesticide distribution patterns across different concentration levels. As shown in Figure 9, the visualization reveals a progressive increase in the detected residue accumulation corresponding to the concentrations of 0%, 0.02%, 0.04%, and 0.06%. At 0%, the perilla leaf surface is blue, indicating absent chlorfenapyr residues. As the concentration increases to 0.02%, 0.04%, and 0.06%, the distribution maps transition to green and yellow, indicating higher residue levels of chlorfenapyr according to the color scale. Due to the non-normal, skewed distribution characteristics of pesticide residues across the perilla leaf surface, non-parametric statistical methods were performed during the significance tests. The Mann–Whitney U test was utilized to evaluate significant differences between consecutive concentration levels [56,57]. The statistical analysis confirmed significant differences (*p* < 0.05) between consecutive concentration levels, validating the visual distinctions observed in the distribution maps. The visualization approach demonstrates the heterogeneous nature of pesticide residue distribution on perilla leaves. It also provides valuable insights into how residue concentrations vary spatially, hence advancing the safety assessment of leafy vegetables.

## 4. Conclusions

This study demonstrates the successful integration of spectral unmixing with short-wavelength infrared hyperspectral imaging (HSI) for non-destructive identification, detection, quantification, and spatial mapping of chlorfenapyr and azoxystrobin residues on perilla leaves. Utilizing multicurve resolution-alternating least squares (MCR-ALS), the method achieved robust residue identification (lack of fit ≤ 1.78%, explained variance > 99%) and enabled detailed spatial visualization of pesticide distributions. Furthermore, combining MCR-ALS insights with Gaussian process regression (GPR) enabled precise quantification of chlorfenapyr concentrations (0–0.06%), yielding a prediction performance with R^2^v = 0.99 and RMSEV = 0.0012%. This novel approach, combining HSI with spectral unmixing for pesticide residue analysis on perilla leaves, highlights the potential for non-destructive, real-time pesticide residue detection in agricultural quality control, ensuring safer produce for consumers. Future studies could perform benchmarking with conventional techniques such as LC–MS, GC–MS, or HPLC, incorporate external datasets, and utilize more samples to establish the reliability, generalizability, and potential of the proposed approach for routine safety in real-world applications. This work paves the way for further research into utilizing spectral unmixing to detect a broader range of contaminants in various agricultural products, contributing to improved food safety standards and sustainable agricultural practices.

## Figures and Tables

**Table 1:** Descriptive statistics of the extracted spectral data for residues on perilla leaves.

Datasets	Mean	Min ^1^	Max ^2^	SD ^3^
	(%)			
Dataset before split	0.030	0	0.060	0.020
Calibration set	0.030	0	0.060	0.022
Test set	0.030	0	0.060	0.023

^1^ Minimum; ^2^ maximum; ^3^ standard deviation.

**Table 2:** Performance parameters for pesticide residues on perilla leaves using the multivariate curve resolution-alternating least squares (MCR-ALS).

Pesticide Residue	Lack of Fit (%)	Variance Explained (%)
chlorfenapyr	1.03	99
azoxystrobin	1.78	99

**Table 3:** Comparison of various regression techniques for estimating chlorfenapyr residue concentration on perilla leaves.

Technique	R^2^c ^1^	RMSEC (%) ^2^	R^2^v ^3^	RMSEV (%) ^4^
MCR-ALS-GPR ^5^	0.99	0.0013	0.99	0.0012
PLSR ^6^	0.98	0.0033	0.97	0.0037
SVR ^7^	0.97	0.0039	0.97	0.0037

^1^ Coefficient of determination of calibration; ^2^ root mean square error of calibration; ^3^ double cross-validation coefficient of determination; ^4^ root mean square error of double cross-validation; ^5^ multivariate curve resolution-alternating least squares combined with Gaussian process regression; ^6^ partial least squares regression; ^7^ support vector regression. The number of observation spectra, n = 120.

## Data Availability

The original contributions presented in the study are included in the article/supplementary material, further inquiries can be directed to the corresponding author.

## Supplementary Materials

The following supporting information can be downloaded at: https://www.mdpi.com/article/10.3390/foods14162864/s1, Figure S1: Perilla leaf samples on a tray before pesticide application; Table S1: Specifications of the main components in the short-wave infrared hyperspectral imaging (SWIR-HSI) system.
