# Supplementary material for: Short-Wavelength Infrared Hyperspectral Imaging and Spectral Unmixing Techniques for Detection and Distribution of Pesticide Residues on Edible Perilla Leaves
Source: Foods. 2025 Aug 18;14(16):2864. doi: 10.3390/foods14162864 (PMC12385282; doi:10.3390/foods14162864)
Supplement: Supplementary file 1 [file foods-14-02864-s001.zip › foods-3778506-supplementary.pdf]

foods

Article

Short-wavelength infrared hyperspectral imaging and spectral unmixing techniques for detection and distribution of pesticide residues on edible perilla leaves

1. Supplementary Material

Table S1. Specifications of the main components in the short-wave infrared hyperspectral imaging (SWIR-HSI) system.

System components and settings	Specifications
Imaging spectrograph	(SWIR, Model number 1003B-10174, Headwall Photonics, Fitchburg, MA, USA) with 894 – 2504 nm wavelength range, and 5.876 nm spectral resolution. Slit width: 25 μm.
Image sensor	A Sterling-cooled mercury cadmium telluride detector array (MCT; HgCdTe, Xeva-2.5-320, Xenics, Belgium) with Pixels: 320 × 256 (Spatial × Spectral channels), Maximum frame rate: 450 Hz.
Light source	Six fiber-optic-coupled 100 W tungsten-halogen lamps (Light Bank, Ushio Inc., Japan). 100Watt- Quartz tungsten halogen (QTH) line light (SWIR via quartz fiber bundles).
Objective Lens	Focal length: 25 mm, f/1.4 (OB-SWIR25/1.4, P/N C0808.010)
Translation stage	Composed of a DC motor, controller, and slide assembly (Xslide, Velmex Inc., Bloomfield, NY, USA).
HSI acquisition settings	Exposure time: 0.043 s, optimal speed: 5.496 mm/s, binning: 1, number of scans: 1.

HSI: hyperspectral imaging, SWIR: short-wave infrared, MCT: mercury cadmium telluride.

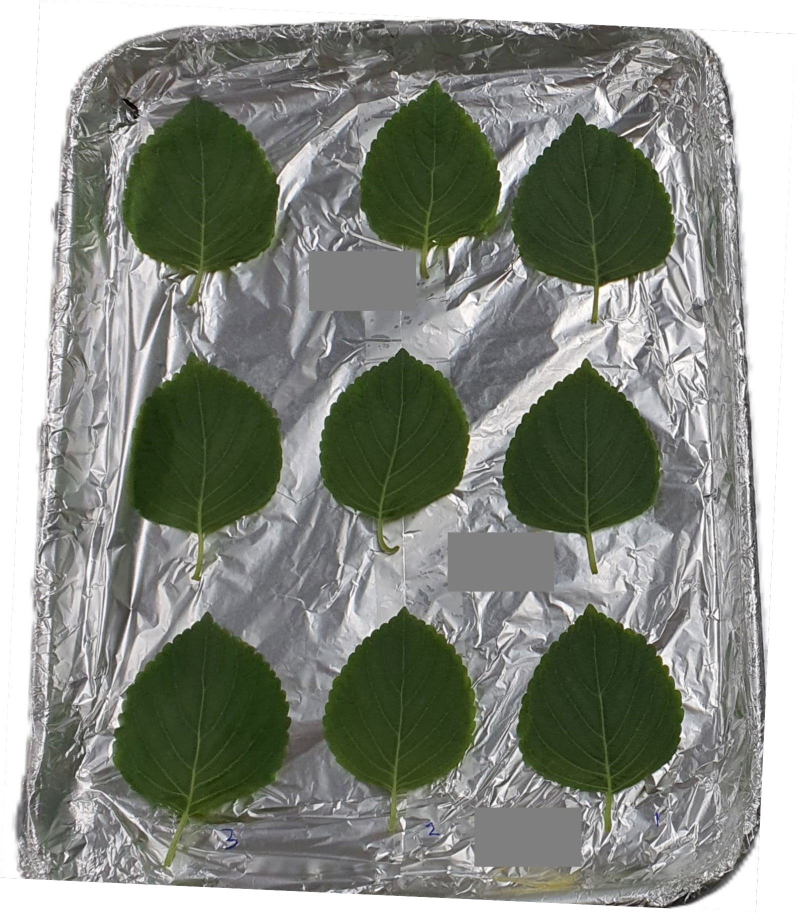

Figure S1. Perilla leaf samples on a tray before pesticide application.
